# Supplementary figures and images for: Photodestruction of Diatomic Molecular Ions: Laboratory and Astrophysical Application
Source: Molecules. 2020 Dec 31;26(1):151. doi: 10.3390/molecules26010151 (PMC7795095; doi:10.3390/molecules26010151)

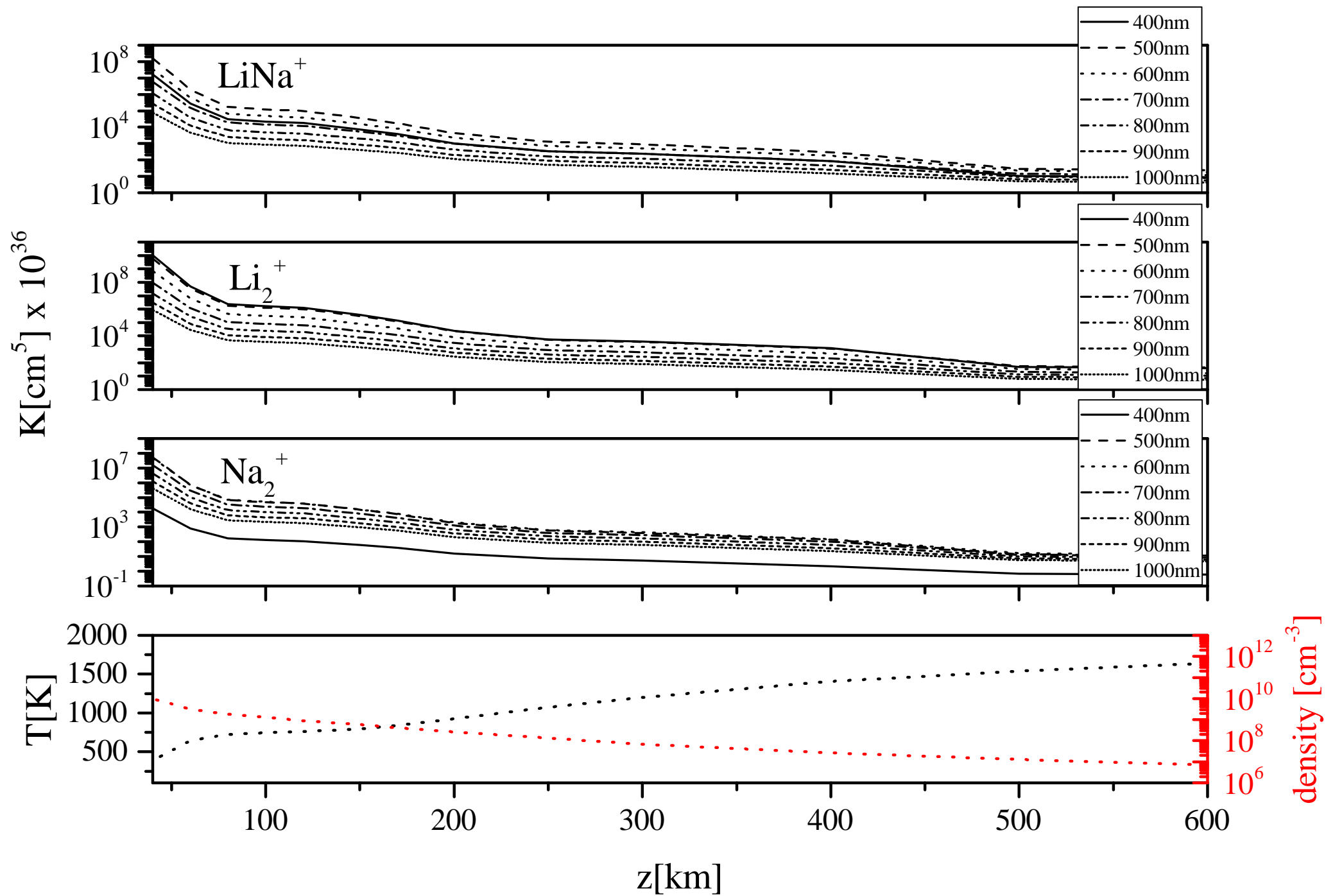

Supplement: Supplementary file 1 [file molecules-26-00151-s001.zip › Io_model_hight_density.pdf]

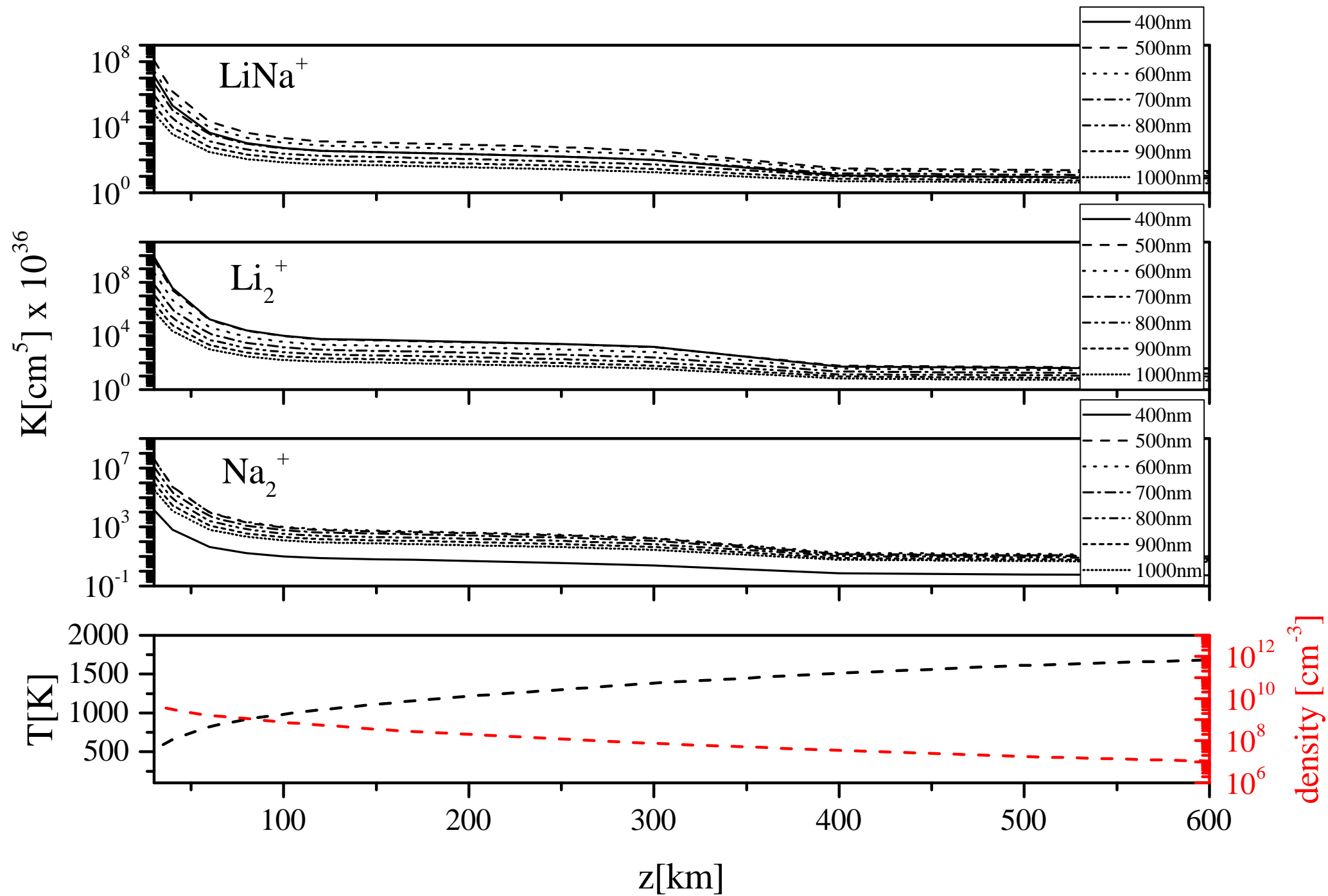

Supplement: Supplementary file 1 [file molecules-26-00151-s001.zip › Io_model_moderat_density.pdf]

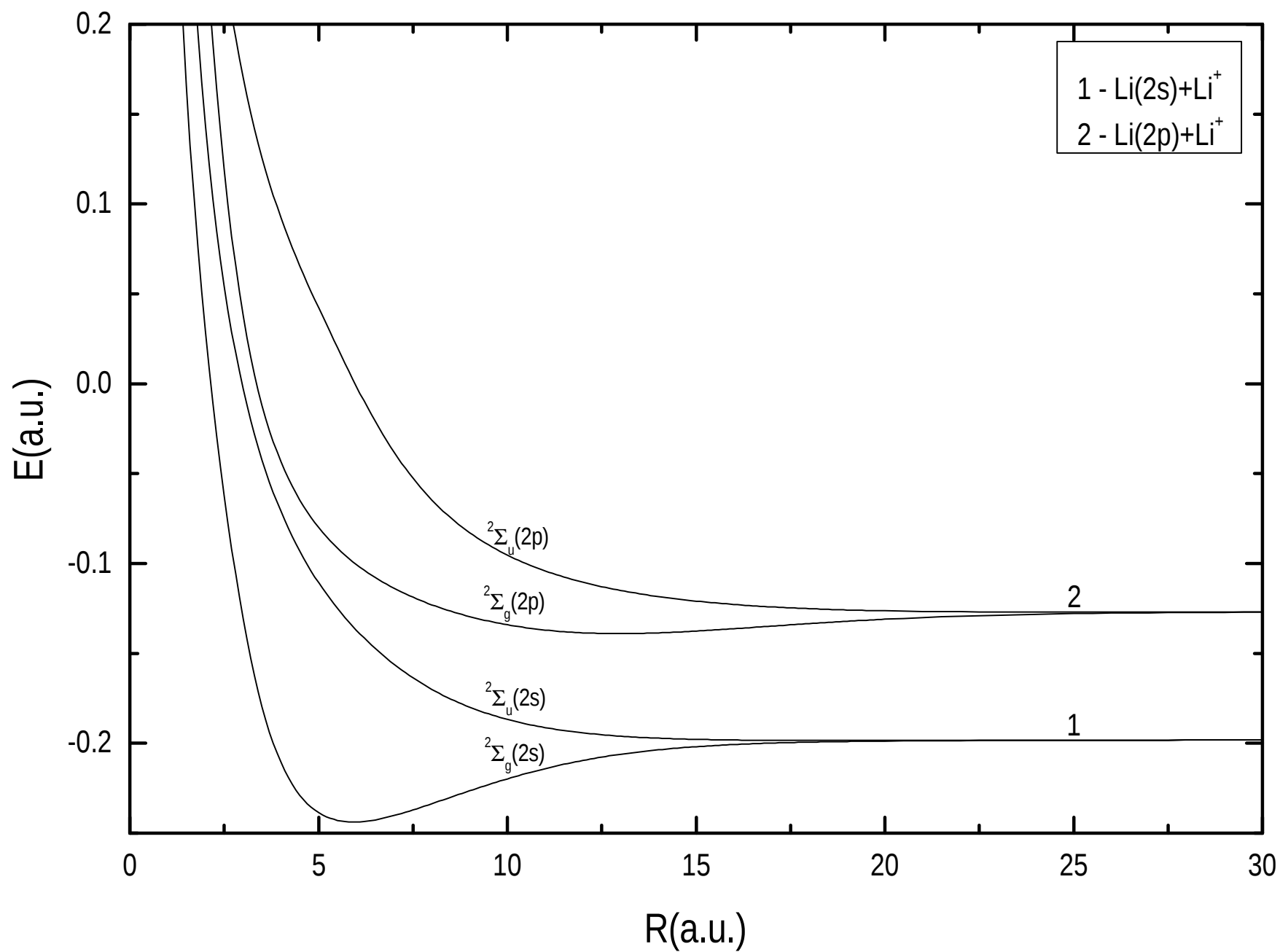

Supplement: Supplementary file 1 [file molecules-26-00151-s001.zip › Li2+.pdf]

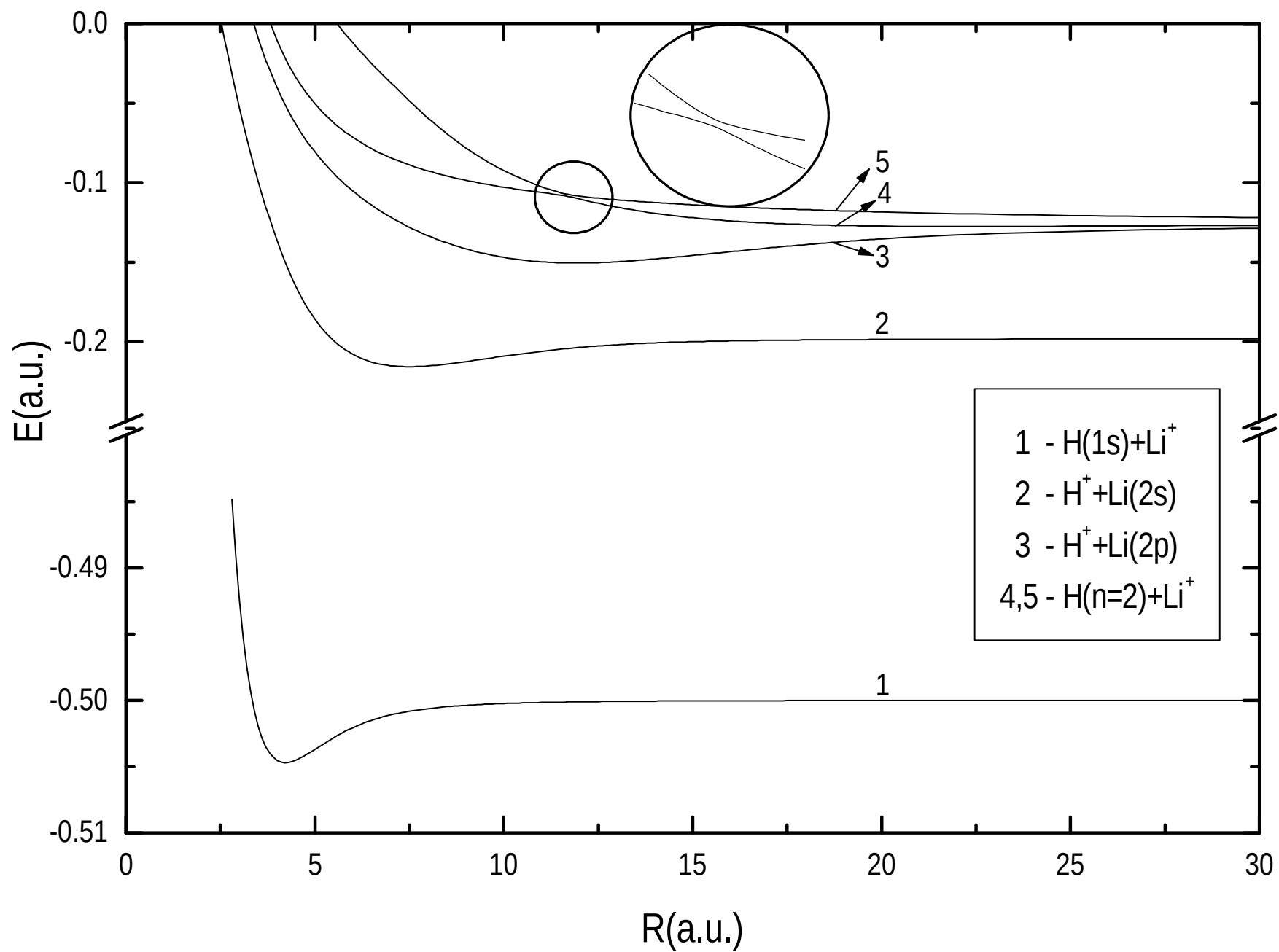

Supplement: Supplementary file 1 [file molecules-26-00151-s001.zip › LiH+.pdf]

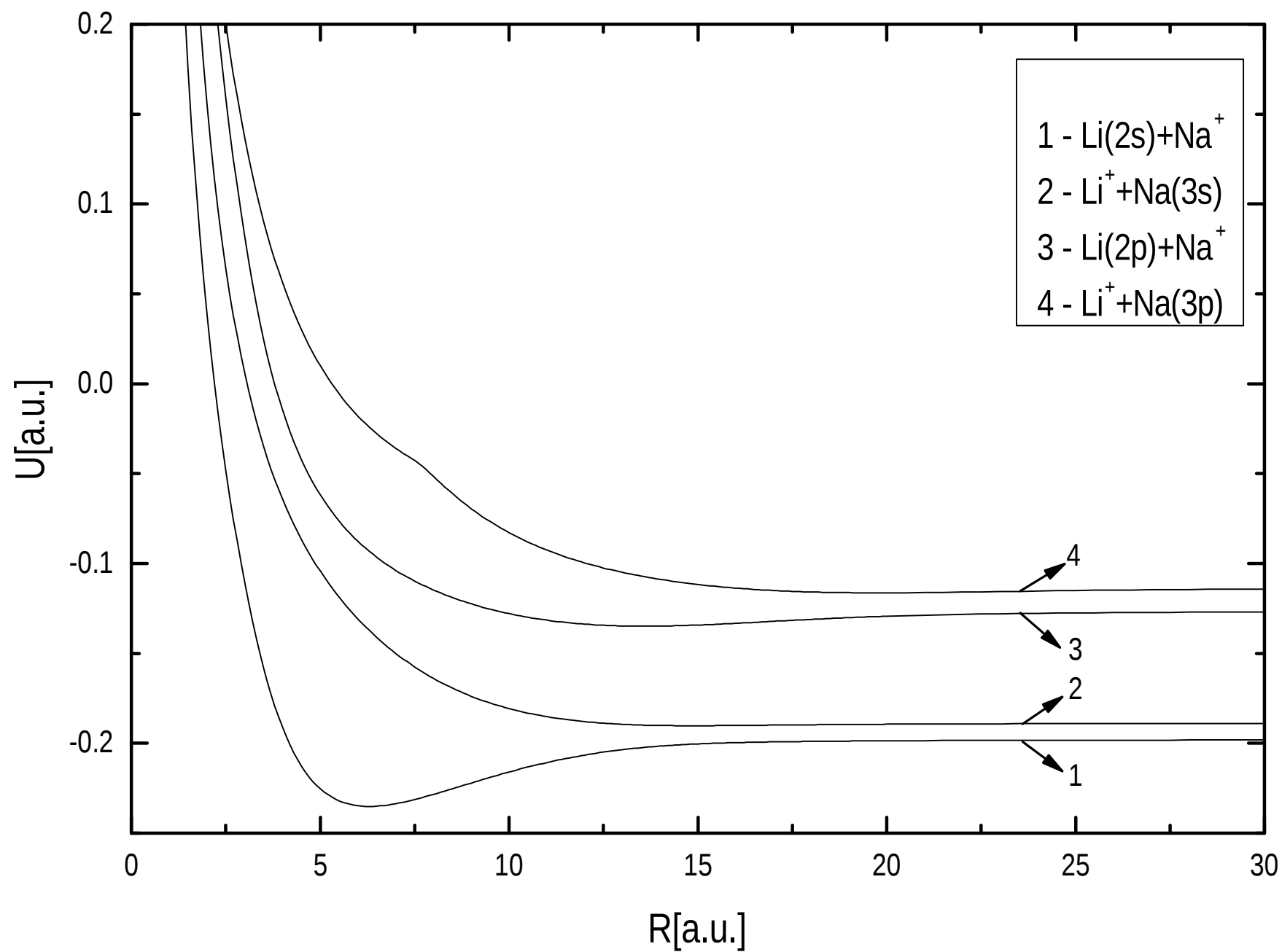

Supplement: Supplementary file 1 [file molecules-26-00151-s001.zip › LiNa+.pdf]

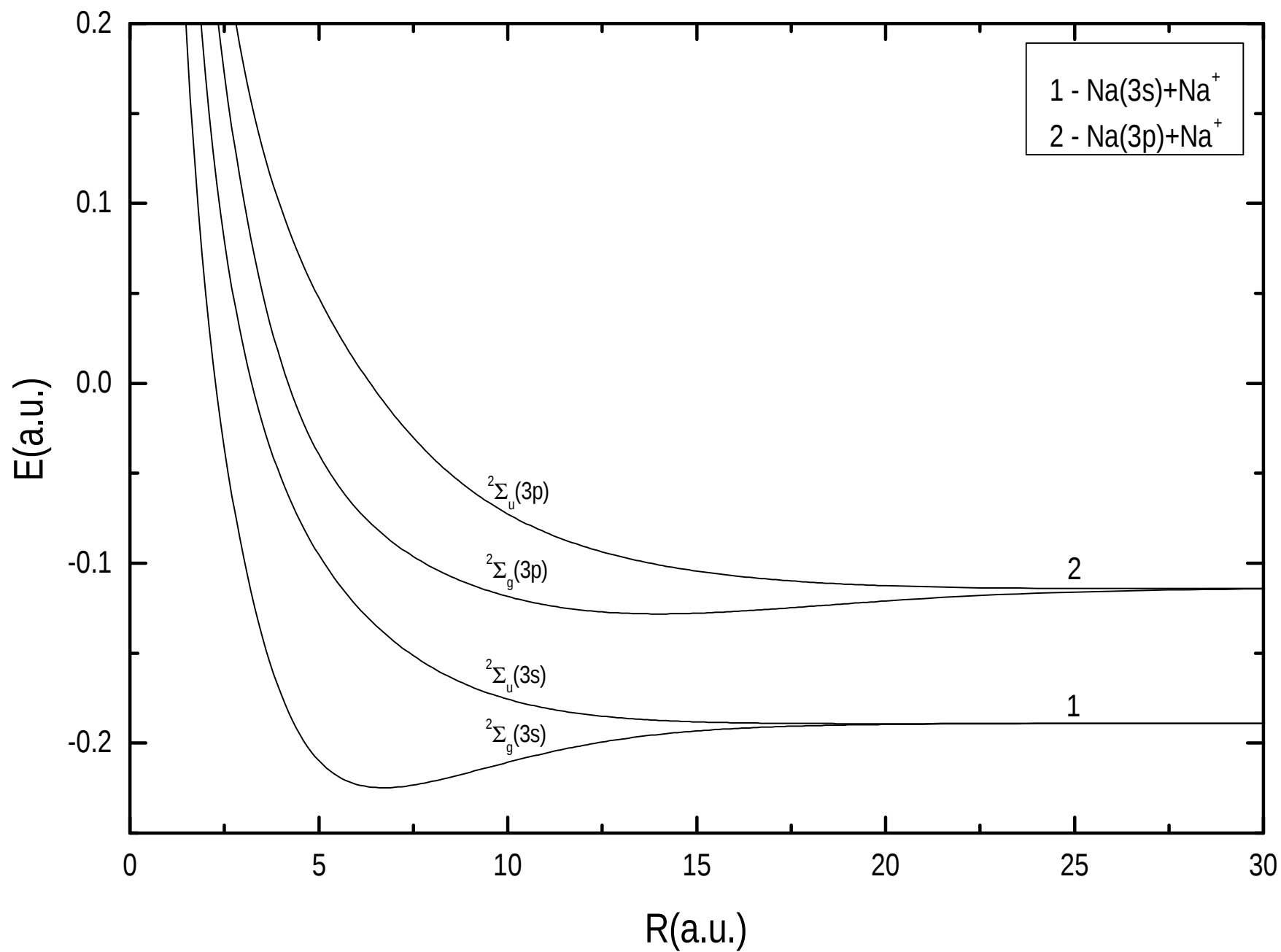

Supplement: Supplementary file 1 [file molecules-26-00151-s001.zip › Na2+.pdf]

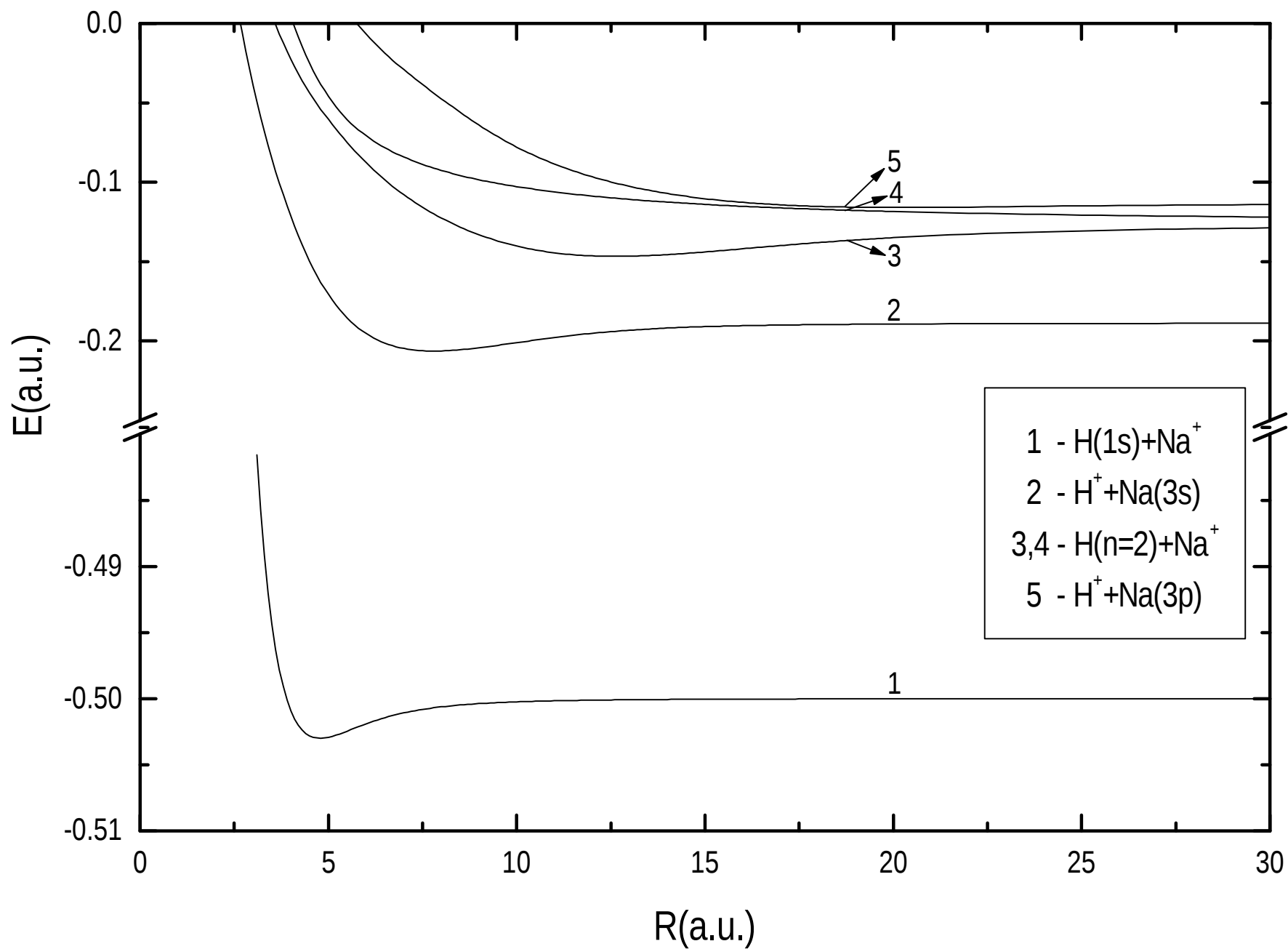

Supplement: Supplementary file 1 [file molecules-26-00151-s001.zip › NaH+.pdf]
